# Rapid maximum likelihood ancestral state reconstruction of continuous characters: A rerooting‐free algorithm

**DOI:** 10.1002/ece3.2837

**Published:** 2017-03-21

**Authors:** Eric W. Goolsby

**Affiliations:** ^1^ Department of Ecology and Evolutionary BiologyBrown UniversityProvidenceRIUSA

**Keywords:** ancestral state reconstruction, fast methods, linear‐time algorithm, maximum likelihood, phylogenetic comparative method, phylogenetic generalized least squares

## Abstract

Ancestral state reconstruction is a method used to study the evolutionary trajectories of quantitative characters on phylogenies. Although efficient methods for univariate ancestral state reconstruction under a Brownian motion model have been described for at least 25 years, to date no generalization has been described to allow more complex evolutionary models, such as multivariate trait evolution, non‐Brownian models, missing data, and within‐species variation. Furthermore, even for simple univariate Brownian motion models, most phylogenetic comparative R packages compute ancestral states via inefficient tree rerooting and full tree traversals at each tree node, making ancestral state reconstruction extremely time‐consuming for large phylogenies. Here, a computationally efficient method for fast maximum likelihood ancestral state reconstruction of continuous characters is described. The algorithm has linear complexity relative to the number of species and outperforms the fastest existing R implementations by several orders of magnitude. The described algorithm is capable of performing ancestral state reconstruction on a 1,000,000‐species phylogeny in fewer than 2 s using a standard laptop, whereas the next fastest R implementation would take several days to complete. The method is generalizable to more complex evolutionary models, such as phylogenetic regression, within‐species variation, non‐Brownian evolutionary models, and multivariate trait evolution. Because this method enables fast repeated computations on phylogenies of virtually any size, implementation of the described algorithm can drastically alleviate the computational burden of many otherwise prohibitively time‐consuming tasks requiring reconstruction of ancestral states, such as phylogenetic imputation of missing data, bootstrapping procedures, Expectation‐Maximization algorithms, and Bayesian estimation. The described ancestral state reconstruction algorithm is implemented in the *Rphylopars* functions *anc.recon* and *phylopars*.

## 1 Introduction

Phylogenetic comparative methods provide a framework for studying phenotypic evolution across species while accounting for statistical nonindependence due to common descent (Felsenstein, 1985; Martins & Hansen, 1997). Ancestral state reconstruction offers a powerful context for studying evolutionary trajectories, such as the number of times a particular phenotype evolved, estimating the approximate timing of major evolutionary events, and inferring missing phenotypic values corresponding to discovered fossils (Garland, Midford, & Ives, 1999; Schluter, Price, Moores, & Ludwig, 1997). Additionally, ancestral reconstruction may help contextualize observed patterns such as correlated shifts between phenotypic and environmental variables. Principles of ancestral state reconstruction may also be used to perform phylogenetic prediction, in which phenotypic values for unobserved or incompletely sampled taxa are estimated based on the evolutionary model and relative phylogenetic position (Garland & Ives, 2000).

Several methods have been developed to reconstruct ancestral phenotypes, including parsimony‐based, Bayesian methods, and maximum likelihood (ML) estimation, the latter of which constitutes the focus of this paper (Felsenstein, 1985; Maddison, 1991; Revell & Reynolds, 2012; Schluter et al., 1997). Like other phylogenetic comparative methods, ancestral state reconstruction becomes increasingly time‐consuming and computationally demanding as the number of species increases. Although efficient algorithms for most applications have existed since the initial development of modern comparative methods, their importance has recently seen a renewed emphasis (FitzJohn, 2012; Freckleton, 2012; Ho & Ané, 2014). Fast comparative methods are critical to keeping up with the ever‐increasing size of phylogenetic trees in studies, as well as for statistical methods requiring thousands or millions of repeated calculations (e.g., parametric bootstrapping, Bayesian inference) (Boettiger & Ralph, 2012; Goolsby, 2016; Hadfield & Nakagawa, 2010; Schluter et al., 1997).

Unlike most comparative methods (e.g., phylogenetic regression, phylogenetic signal, estimation of alternative evolutionary models), computationally efficient methods for performing ancestral state reconstruction are severely lacking. This is because, despite the existence of efficient comparative methods that avoid the need to invert the phylogenetic covariance matrix, most R implementations of ML ancestral state reconstruction rely on (1) rerooting the tree at each internal node and performing repeated calculations (Revell, 2012), (2) high‐dimensional numerical optimization (Paradis, Claude, & Strimmer, 2004), or (3) parameterizing and manipulating extremely large covariance matrices (Ho & Ané, 2014; Paradis et al., 2004).

This paper introduces a computationally efficient, generalizable, two‐pass algorithm for performing ML ancestral state reconstruction which outperforms existing implementations by several orders of magnitude. The algorithm is first described in univariate terms and is mathematically identical to efficient algorithms described by Maddison (1991), Felsenstein (2004), and Elliot (2015). Next, the algorithm is generalized to multivariate trait evolution, non‐Brownian models, missing data, and within‐species variation.

The first pass of the algorithm is identical to the linear‐time algorithm described in Ho and Ané (2014), which computes quantities at the root of the tree using a postorder (tips to root) tree traversal algorithm. The second pass of the algorithm operates by holding values computed at the root constant and recursively traversing the tree in preorder (root to tips) to compute quantities of interest at each internal node. The algorithm is implemented in the R package *Rphylopars* in the functions *anc.recon* and *phylopars* (Goolsby, Bruggeman, & Ané, 2017).

## 2 Methods

### 2.1 Fast algorithm for ML ancestral state reconstruction

Here, we define a two‐pass (postorder–preorder) recursive algorithm for calculating several quantities of interest related to ML ancestral state reconstruction at each node of the tree. The postorder portion of the algorithm as described in Ho and Ané (2014) partitions the phylogeny into recursively defined subtrees. For a terminal node (a tip) on the tree, the corresponding subtree consists of a single node (i.e., the tip of the subtree is also the root of the subtree), and the edge giving rise to the tip on the original phylogeny is the root edge of the subtree. For a bifurcating internal node, the corresponding subtree has two tips and a single internal node with a root edge (for a polytomous internal node, the subtree has multiple tips and a root edge). Like the PIC algorithm (Felsenstein, 1985), the postorder portion of the algorithm recursively computes locally parsimonious values for quantities of interest, including the expected variance due to phylogeny and estimated ancestral states at each internal node (Ho & Ané, 2014). In other words, local quantities that are calculated for a given node represent the global quantities that would be obtained if the tree consisted only of the given node and its descendants. At the root of the original phylogeny, the computed local quantity is equivalent to the global quantity, corresponding to globally parsimonious and maximum likelihood estimates (Felsenstein, 1985; Garland et al., 1999; Ho & Ané, 2014; Maddison, 1991). Conversely, the local quantities obtained for all other internal nodes are *not* global quantities because they do not account for the information contained in the rest of the phylogeny. However, because the postorder algorithm computes global quantities for the root of the tree, we can hold the root quantities constant and solve for values at its descendent nodes, which can then themselves be held constant to solve for their descendent nodes, and so on, until we reach the tips of the tree. The two‐pass algorithm is mathematically equivalent to rerooting strategies for obtaining global estimates for each node (which are the current method‐of‐choice for rapid ancestral state reconstruction in R (Revell, 2012)), but the proposed algorithm avoids redundant time‐consuming operations and is accordingly several orders of magnitude faster.

The two‐pass algorithm described here computes the following quantities: ${\hat{\mu }}^{\left(e\right)}={\left(1^{\prime }C^{{\left(e\right)}^{-1}}1\right)}^{-1}1^{\prime }C^{{\left(e\right)}^{-1}}Y,\;\;p^{\left(e\right)}=1^{\prime }C^{{\left(e\right)}^{-1}}1,\;Q^{\left(e\right)}=L^{\prime }C^{{\left(e\right)}^{-1}}R$, and the log determinant of the species covariance matrix (log|**C** ^(*e*)^|), where **1** is a vector of ones, ${\hat{\mu }}^{\left(e\right)}$ is the ML ancestral estimate for **Y** at the node arising from edge *e*,** C** ^(*e*)^ is the species covariance matrix obtained by rerooting the phylogeny at the node arising from edge *e*, and **L** and **R** are matrices of compatible dimensions in the product $L^{\prime }C^{{\left(e\right)}^{-1}}R$ (e.g., **L** = **1** and **R** = **Y**). These quantities are computed via preorder tree traversal following postorder computation of the local quantities ${\tilde{\mu }}^{\left(e\right)}={\left(1^{\prime }{\tilde{C}}^{{\left(e\right)}^{-1}}1\right)}^{-1}1^{\prime }{\tilde{C}}^{{\left(e\right)}^{-1}}Y$, ${\tilde{p}}^{\left(e\right)}=1^{\prime }{\tilde{C}}^{{\left(e\right)}^{-1}}1,\;{\tilde{Q}}^{\left(e\right)}=L^{\prime }{\tilde{C}}^{{\left(e\right)}^{-1}}R$, and $\log|{\tilde{C}}^{\left(e\right)}|$, where ${\tilde{C}}^{\left(e\right)}$ is the species covariance matrix obtained by pruning the tree to only the descendants arising from (but not including) edge *e*. Note that $1/{\tilde{p}}^{\left(e\right)}$ is equivalent to the transformed branch lengths obtained using the phylogenetically independent contrasts (PIC) algorithm and ${\tilde{\mu }}^{\left(e\right)}$ is equivalent to PIC‐based (locally parsimonious) ancestral state reconstruction (Felsenstein, 1985).

1. Initialization: for edge *e* of length *t* ^(*e*)^ giving rise to a terminal taxon, define as follows:

$${\tilde{\mu }}^{\left(e\right)}=y^{\left(e\right)}$$

$${\tilde{p}}^{\left(e\right)}=1/t^{\left(e\right)}$$

$${\tilde{U}}^{{\left(e\right)}^{\prime }}=L^{{\left(e\right)}^{\prime }}/t^{\left(e\right)}$$

$${\tilde{V}}^{\left(e\right)}=R^{\left(e\right)}/t^{\left(e\right)}$$

$${\tilde{Q}}_{e}=L^{{\left(e\right)}^{\prime }}R^{\left(e\right)}/t^{\left(e\right)}$$

$$\log|{\tilde{C}}^{\left(e\right)}|=\log\left(t^{\left(e\right)}\right)$$

Postorder recursion: for edge *e* of length *t* ^(*e*)^ giving rise to an internal node, define for all immediate descendants (*d*) of edge *e*:

$$p_{A}^{\left(e\right)}=\Sigma {\tilde{p}}^{\left(d\right)}$$

$${\tilde{\mu }}^{\left(e\right)}=\Sigma \left({\tilde{\mu }}^{\left(d\right)}{\tilde{p}}^{\left(d\right)}/p_{A}^{\left(e\right)}\right)$$

$${\tilde{p}}^{\left(e\right)}=p_{A}^{\left(e\right)}/\left(1+t^{\left(e\right)}p_{A}^{\left(e\right)}\right)$$

$${\tilde{U}}^{{\left(e\right)}^{\prime }}=\left(\Sigma {\tilde{U}}^{{\left(d\right)}^{\prime }}\right)/\left(1+t^{\left(e\right)}p_{A}^{\left(e\right)}\right)$$

$${\tilde{V}}^{\left(e\right)}=\left(\Sigma {\tilde{V}}^{\left(d\right)}\right)/\left(1+t^{\left(e\right)}p_{A}^{\left(e\right)}\right)$$

$${\tilde{Q}}^{\left(e\right)}=\Sigma {\tilde{Q}}^{\left(d\right)}-\left(\Sigma {\tilde{U}}^{{\left(d\right)}^{\prime }}\right)\left(\Sigma {\tilde{V}}^{\left(d\right)}\right)t^{\left(e\right)}/\left(1+t^{\left(e\right)}p_{A}^{\left(e\right)}\right)$$

$$\log|{\tilde{C}}_{e}\left|=\Sigma \log\right|{\tilde{C}}^{\left(d\right)}|+\log\left(1+t^{\left(e\right)}p_{A}^{\left(e\right)}\right)$$

At the root edge of the tree, denote as follows:

$${\hat{\mu }}^{\left(r\right)}={\tilde{\mu }}^{\left(r\right)}$$

$$p^{\left(r\right)}={\tilde{p}}^{\left(r\right)}$$

$$U^{{\left(r\right)}^{\prime }}={\tilde{U}}^{{\left(r\right)}^{\prime }}$$

$$V^{\left(r\right)}={\tilde{V}}^{\left(r\right)}$$

$$Q^{\left(r\right)}={\tilde{Q}}^{\left(r\right)}$$

$$\log|C^{\left(r\right)}\left|=\log\right|{\tilde{C}}^{\left(r\right)}|$$

Preorder recursion: for edge *e* (which arises from the node arising from ancestral edge *a*) of length *t*_*e*_ giving rise to an internal node, define as follows:

$${\hat{\mu }}^{\left(e\right)}={\tilde{\mu }}^{\left(e\right)}{\tilde{p}}^{\left(e\right)}t^{\left(e\right)}+{\hat{\mu }}^{\left(a\right)}-{\hat{\mu }}^{\left(a\right)}{\tilde{p}}^{\left(e\right)}t^{\left(e\right)}$$

$$p^{\left(e\right)}={\tilde{p}}^{\left(e\right)}/\left(1-t^{\left(e\right)}{\tilde{p}}^{\left(e\right)}\right)+\left(p^{\left(a\right)}-{\tilde{p}}^{\left(e\right)}\right)/\left(1+t^{\left(e\right)}\left(p^{\left(a\right)}-{\tilde{p}}^{\left(e\right)}\right)\right)$$

$$U^{{\left(e\right)}^{\prime }}={\tilde{U}}^{{\left(e\right)}^{\prime }}/\left(1-t^{\left(e\right)}{\tilde{p}}^{\left(e\right)}\right)+\left(U^{{\left(a\right)}^{\prime }}-{\tilde{U}}^{{\left(e\right)}^{\prime }}\right)/\left(1+t^{\left(e\right)}\left(p^{\left(a\right)}-{\tilde{p}}^{\left(e\right)}\right)\right)$$

$$V^{\left(e\right)}={\tilde{V}}^{\left(e\right)}/\left(1-t^{\left(e\right)}{\tilde{p}}^{\left(e\right)}\right)+\left(V^{\left(a\right)}-{\tilde{V}}^{\left(e\right)}\right)/\left(1+t^{\left(e\right)}\left(p^{\left(a\right)}-{\tilde{p}}^{\left(e\right)}\right)\right)$$

$$\begin{array}{cc} & Q^{\left(e\right)}=\left[{\tilde{Q}}^{\left(e\right)}-\left({\tilde{U}}^{{\left(e\right)}^{\prime }}\right)\left({\tilde{V}}^{\left(e\right)}\right)\left(-t^{\left(e\right)}\right)/\left(1-t^{\left(e\right)}{\tilde{p}}^{\left(e\right)}\right)\right]+\\ & \;\left[\left(Q^{\left(a\right)}-{\tilde{Q}}^{\left(e\right)}\right)-\left(U^{{\left(a\right)}^{\prime }}-{\tilde{U}}^{{\left(e\right)}^{\prime }}\right)\left(V^{\left(a\right)}-{\tilde{V}}^{\left(e\right)}\right)t^{\left(e\right)}/\left(1+t^{\left(e\right)}\left(p^{\left(a\right)}-{\tilde{p}}^{\left(e\right)}\right)\right)\right] \end{array}$$

$$\log|C^{\left(e\right)}\left|=\log\right|C^{\left(a\right)}|+\log\left(1-t^{\left(e\right)}{\tilde{p}}^{\left(e\right)}\right)+\log\left(1+t^{\left(e\right)}\left(p^{\left(a\right)}-{\tilde{p}}^{\left(e\right)}\right)\right)$$

For a linear regression model, we may also compute the regression parameters ${\hat{\beta }}^{\left(e\right)}={\left(Q_{\mathrm{XX}}^{\left(e\right)}\right)}^{-1}Q_{\mathrm{XY}}^{\left(e\right)}$ where **X** is a design matrix (for an intercept‐only model, **X **=** 1** as above; for a regression model, the first column typically consists of ones and the remaining columns consist of values for predictor variables).

Ho and Ané (2014) proved that the postorder recursion algorithm yields the global quantities ${\hat{\mu }}^{\left(r\right)},\;p^{\left(r\right)},\;Q^{\left(r\right)}$, and log | **C** ^(*r*)^ |, and it has been long‐established that rerooting the tree yields global estimates of these quantities for any node of the tree (Garland & Ives, 2000; Maddison, 1991; Swofford & Maddison, 1987). The preorder recursion step works by mathematically rerooting each subtree recursively at each node. To demonstrate the properties of the preorder recursion, first consider that the original phylogeny lacks a root edge (*t* ^(*r*)^ = 0), so step 3 reduces to $p^{\left(r\right)}=\Sigma {\tilde{p}}^{\left(d\right)}$. Therefore, we may treat the current subtree as being composed of two descendent edges which we denote *e* and *other,* such that $p^{\left(r\right)}=\Sigma {\tilde{p}}^{\left(d\right)}={\tilde{p}}^{\left(e\right)}+{\tilde{p}}^{\left(\mathrm{other}\right)}$, which can also be expressed as $p^{\left(r\right)}={\tilde{p}}^{\left(e\right)}+\left(p^{\left(r\right)}-{\tilde{p}}^{\left(e\right)}\right)$ to avoid having to keep track of ${\tilde{p}}^{\left(\mathrm{other}\right)}$ (note that this holds true even if the subtree arising from *other* were in fact polytomous).

To compute the quantity *p* ^(*e*)^, we could reroot the original tree at the node arising from edge *e* and then perform steps 1–3 of the postorder algorithm (Garland et al., 1999; Ho & Ané, 2014). However, the majority of these steps would be redundant, as we have already computed all of these quantities up to our node of interest. To see this, note that had the original tree been rooted at the node arising from edge *e* rather than *r*, the original computation for ${\tilde{p}}^{\left(e\right)}$ would have been ${\tilde{p}}^{{\left(e\right)}^{*}}=p_{A}^{\left(e\right)}$ instead of ${\tilde{p}}^{\left(e\right)}=p_{A}^{\left(e\right)}/\left(1+t^{\left(e\right)}p_{A}^{\left(e\right)}\right)$ because *t* ^(*e*)^ would have equaled zero (the length of *t* ^(*e*)^ would have instead been added to the length of *t* ^(other)^). To adjust for this, we cancel out the contribution of *t* ^(*e*)^ as follows: ${\tilde{p}}^{{\left(e\right)}^{*}}={\tilde{p}}^{\left(e\right)}/\left(1-t^{\left(e\right)}{\tilde{p}}^{\left(e\right)}\right)$. Now, we must add the contribution of *t* ^(*e*)^ to ${\tilde{p}}^{\left(\mathrm{other}\right)}$, as follows: ${\tilde{p}}^{{\left(\mathrm{other}\right)}^{*}}={\tilde{p}}^{\left(\mathrm{other}\right)}/\left(1+t^{\left(e\right)}{\tilde{p}}^{\left(\mathrm{other}\right)}\right)=\left(p^{\left(r\right)}-{\tilde{p}}^{\left(e\right)}\right)/\left(1+t^{\left(e\right)}\left(p^{\left(r\right)}-{\tilde{p}}^{\left(e\right)}\right)\right)$. Therefore, we have now obtained the quantities necessary to compute $p^{{\left(r\right)}^{*}}$ (i.e., had the tree been rooted at the node arising from edge *e*) without actually having to reroot the tree or perform any redundant calculations: $p^{\left(e\right)}={\tilde{p}}^{{\left(e\right)}^{*}}+{\tilde{p}}^{{\left(\mathrm{other}\right)}^{*}}={\tilde{p}}^{\left(e\right)}/\left(1-t^{\left(e\right)}{\tilde{p}}^{\left(e\right)}\right)+\left(p^{\left(a\right)}-{\tilde{p}}^{\left(e\right)}\right)/\left(1+t^{\left(e\right)}\left(p^{\left(a\right)}-{\tilde{p}}^{\left(e\right)}\right)\right)$. The same procedure immediately follows for the computations of $U^{{\left(e\right)}^{\prime }}$, **V** ^(*e*)^, **Q** ^(*e*)^, and log | **C** ^(*e*)^ |  because at the root of the tree, these quantities are simply composed of the sums of their descendant quantities (because *t* ^(*r*)^ = 0). The ancestral state reconstruction ${\hat{\mu }}^{\left(e\right)}$ is an algebraic simplification of the quantity ${\hat{\mu }}^{\left(e\right)}={\left(Q_{11}^{\left(e\right)}\right)}^{-1}Q_{1Y}^{\left(e\right)}$ where $Q_{11}^{\left(e\right)}=1^{\prime }C^{{\left(e\right)}^{-1}}1$ and $Q_{1Y}^{\left(e\right)}=1^{\prime }C^{{\left(e\right)}^{-1}}Y$. By repeating step 4 recursively from the root to the tips, we obtain global ML estimates for each internal node.

The covariance of a given estimate can be computed as follows: ${\text{cov}}_{{\hat{\mu }}^{\left(e\right)}}=\hat{\Sigma }/p^{\left(e\right)}$ where $\hat{\Sigma }$ is the ML or restricted ML evolutionary rate matrix: $\hat{\Sigma }=\left({\left(Y-1{\hat{\mu }}^{\left(r\right)}\right)}^{\prime }C^{{\left(r\right)}^{-1}}\left(Y-1{\hat{\mu }}^{\left(r\right)}\right)\right)/\left(N-\text{REML}\right)$, *N* is the number of species, and REML = 1 if the restricted ML estimate is desired and REML = 0 otherwise. 95% confidence intervals for an estimate can then be computed as follows: $95\%\;{\text{C.I.}}_{{\hat{\mu }}^{\left(e\right)}}={\hat{\mu }}^{\left(e\right)}\pm 1.96\sqrt{{\text{var}}_{{\hat{\mu }}^{\left(e\right)}}}$ (Garland & Ives, 2000; Garland et al., 1999; Rohlf, 2001).

### 2.2 Multivariate data, alternative evolutionary models, within‐species variation, and missing data

The described algorithm can be easily modified to incorporate a wide variety of models with various features such as missing data, intraspecific variation, and alternative evolutionary model specifications (Bruggeman, Heringa, & Brandt, 2009; Felsenstein, 2008; Goolsby et al., 2017; Ives, Midford, & Garland, 2007). For a multivariate model of evolution, the N × M matrix **Y** (where *M* is the number of traits) is rearranged into an *NM*‐length column vector **y**, the matrix **1** is replaced with an NM × M matrix describing which observations of **y** are from which trait, and the covariance of each observation is described by an NM × NM species‐trait covariance matrix **W**. For a Brownian motion model of evolution, **W** = **Σ** ⊗ **C**, where **Σ** is the evolutionary rate matrix, ⊗  denotes the Kronecker product, and **W** is partitioned into *M* ^2^ blocks of size N × N. For example, at block *i*,* j*,** W**_*i*,*j*_ = *Σ*_*i*,*j*_ **C**. When considering the node arising from a single edge *e*, we are left with an M × M matrix of transformed heights (root‐to‐node distance): **H** ^(*e*)^ = *C*_*a*,*b*_ **Σ**, and the node arising form edge *e* is the most recent common ancestor of species *a* and *b*. The height matrix **H**_*e*_ can be converted into an edge length matrix **T** ^(*e*)^ as follows: **T** ^(*e*)^ = **H** ^(*a*)^ − **H** ^(*e*)^ (which also equals *t* ^(*e*)^ **Σ** for a Brownian motion model), where the node arising from edge *a* is the parent of edge *e*. For Brownian motion models, we can simply use **T** ^(*e*)^ = *t* ^(*e*)^ **Σ**. To accommodate rate shift models, the estimated regime‐specific rate matrices **Σ** ^(*s*)^ may be used: **T** ^(*e*)^ = *t* ^(*e*)^ **Σ** ^(*s*)^. For more complex evolutionary models (e.g., multivariate Ornstein–Uhlenbeck on an ultrametric tree), **W** is scaled according to block‐specific transformations, and we must compute **T** ^(*e*)^ = **H** ^(*a*)^ − **H** ^(*e*)^ for each edge (for a derivation, see Goolsby et al., 2017; Appendix S1). It should be noted that the algorithm requires an ultrametric tree if an Ornstein–Uhlenbeck model is specified; otherwise, a complex series of branch length and data transformations must be made to maintain three‐point structure as described in Ho and Ané (2014). The multivariate algorithm proceeds as follows:

1. Initialization: for edge *e* with length matrix **T** ^(*e*)^ giving rise to a terminal taxon, for the subset of variables **k** on which data are available (nonmissing)

$${\tilde{p}}_{k,k}^{\left(e\right)}=T_{k,k}^{{\left(e\right)}^{-1}}$$

${\tilde{U}}_{k}^{{\left(e\right)}^{\prime }}=L_{k}^{{\left(e\right)}^{\prime }}{\tilde{p}}_{k,k}^{\left(e\right)}$ for the subset of variables on which data are available. Rows of ${\tilde{U}}^{\left(e\right)}$ corresponding to missing data are set to zero.

${\tilde{V}}_{k}^{\left(e\right)}={\tilde{p}}_{k,k}^{\left(e\right)}R_{k}^{\left(e\right)}$ for the subset of variables on which data are available. Columns of ${\tilde{V}}^{\left(e\right)}$ corresponding to variables with missing data are set to zero.

${\tilde{Q}}_{k,k}^{\left(e\right)}=L_{k}^{{\left(e\right)}^{\prime }}{\tilde{p}}_{k,k}^{\left(e\right)}R_{k}^{\left(e\right)}$ for the subset of variables on which data are available. Rows and columns of ${\tilde{Q}}^{\left(e\right)}$ corresponding to missing data are set to zero.

$\log|{\tilde{W}}^{\left(e\right)}\left|=\log\right|T_{k,k}^{\left(e\right)}|$ for the subset of variables on which data are available.

Postorder recursion: for edge *e* with length matrix **T** ^(*e*)^ giving rise to an internal node, define for all immediate descendants (*d*) of edge *e*:

$$p_{A}^{\left(e\right)}=\Sigma {\tilde{p}}^{\left(d\right)}$$

$${\tilde{p}}^{\left(e\right)}=p_{A}^{\left(e\right)}{\left(I+T^{\left(e\right)}p_{A}^{\left(e\right)}\right)}^{-1}$$

$${\tilde{U}}^{{\left(e\right)}^{\prime }}=\left(\Sigma {\tilde{U}}^{{\left(d\right)}^{\prime }}\right){\left(I+T^{\left(e\right)}p_{A}^{\left(e\right)}\right)}^{-1}$$

$${\tilde{V}}^{\left(e\right)}={\left({\left(\Sigma {\tilde{V}}^{\left(d\right)}\right)}^{\prime }{\left(I+T^{\left(e\right)}p_{A}^{\left(e\right)}\right)}^{-1}\right)}^{\prime }$$

$${\tilde{Q}}^{\left(e\right)}=\Sigma {\tilde{Q}}^{\left(d\right)}-\left(\Sigma {\tilde{U}}^{\left(d\right)}\right){\left(I+T^{\left(e\right)}p_{A}^{\left(e\right)}\right)}^{-1}T^{\left(e\right)}\left(\Sigma {\tilde{V}}^{\left(d\right)}\right)$$

$$\log|{\tilde{W}}^{\left(e\right)}\left|=\Sigma \log\right|{\tilde{W}}^{\left(d\right)}|+\log|I+T^{\left(e\right)}p_{A}^{\left(e\right)}|$$

At the root edge of the tree, denote:

$$p^{\left(r\right)}={\tilde{p}}^{\left(r\right)}$$

$$U^{{\left(r\right)}^{\prime }}={\tilde{U}}^{{\left(r\right)}^{\prime }}$$

$$V^{\left(r\right)}={\tilde{V}}^{\left(r\right)}$$

$$Q^{\left(r\right)}={\tilde{Q}}^{\left(r\right)}$$

$$\log|W^{\left(r\right)}\left|=\log\right|{\tilde{W}}^{\left(e\right)}|$$

$${\hat{\mu }}^{\left(r\right)}={\left(Q_{11}^{\left(r\right)}\right)}^{-1}Q_{1Y}^{\left(r\right)}$$

Preorder recursion: for edge *e* (which arises from the node arising from ancestral edge *a*) of length **T** ^(*e*)^ giving rise to an internal node, define

$$p^{\left(e\right)}={\tilde{p}}^{\left(e\right)}{\left(I-T^{\left(e\right)}{\tilde{p}}^{\left(e\right)}\right)}^{-1}+\left(p^{\left(a\right)}-{\tilde{p}}^{\left(e\right)}\right){\left(I+T^{\left(e\right)}\left(p^{\left(a\right)}-{\tilde{p}}^{\left(e\right)}\right)\right)}^{-1}$$

$$U^{{\left(e\right)}^{\prime }}={\tilde{U}}^{{\left(e\right)}^{\prime }}{\left(I-T^{\left(e\right)}{\tilde{p}}^{\left(e\right)}\right)}^{-1}+\left(U^{{\left(a\right)}^{\prime }}-{\tilde{U}}^{{\left(e\right)}^{\prime }}\right){\left(I+T^{\left(e\right)}\left(p^{\left(a\right)}-{\tilde{p}}^{\left(e\right)}\right)\right)}^{-1}$$

$$V^{\left(e\right)}={\left[{\tilde{V}}^{{\left(e\right)}^{\prime }}{\left(I-T^{\left(e\right)}{\tilde{p}}^{\left(e\right)}\right)}^{-1}+{\left(V^{\left(a\right)}-{\tilde{V}}^{\left(e\right)}\right)}^{\prime }{\left(I+T^{\left(e\right)}\left(p^{\left(a\right)}-{\tilde{p}}^{\left(e\right)}\right)\right)}^{-1}\right]}^{\prime }$$

$$\begin{array}{cc} & Q^{\left(e\right)}=\left[{\tilde{Q}}^{\left(e\right)}-\left({\tilde{U}}^{{\left(e\right)}^{\prime }}\right)\left({\tilde{V}}^{\left(e\right)}\right)\left(-T^{\left(e\right)}\right)\right]{\left(I-T^{\left(e\right)}{\tilde{p}}^{\left(e\right)}\right)}^{-1}+\\ & \;\left[\left(Q^{\left(a\right)}-{\tilde{Q}}^{\left(e\right)}\right)-\left(U^{{\left(a\right)}^{\prime }}-{\tilde{U}}^{{\left(e\right)}^{\prime }}\right){\left(I+T^{\left(e\right)}\left(p^{\left(a\right)}-{\tilde{p}}^{\left(e\right)}\right)\right)}^{-1}T^{\left(e\right)}\left(V^{\left(a\right)}-{\tilde{V}}^{\left(e\right)}\right)\right] \end{array}$$

$$\log|W_{e}\left|=\log\right|W_{a}|+\log|I-T^{\left(e\right)}{\tilde{p}}^{\left(e\right)}|+\log|I+T^{\left(e\right)}\left(p^{\left(a\right)}-{\tilde{p}}^{\left(e\right)}\right)|$$

$${\hat{\mu }}^{\left(e\right)}={\left(Q_{11}^{\left(e\right)}\right)}^{-1}Q_{1Y}^{\left(e\right)}$$

For edge *e* (which arises from the node arising from ancestral edge *a*) of length **T** ^(*e*)^ giving rise to a terminal node (a tip) with missing data on a subset of variables (**u**) and nonmissing data for subset **k**, define

$${\hat{\mu }}_{u}^{\left(e\right)}=T_{u,k}^{\left(e\right)}T_{k,k}^{{\left(e\right)}^{-1}}{\left(y_{k}^{\left(e\right)}-{\hat{\mu }}_{k}^{\left(a\right)}\right)}^{\prime }+{\hat{\mu }}_{u}^{\left(a\right)}$$

$${\text{cov}}_{{\hat{\mu }}_{u}^{\left(e\right)}}={\left[\left(p^{\left(a\right)}-{\tilde{p}}^{\left(e\right)}\right){\left(I+T^{\left(e\right)}{\tilde{p}}^{\left(e\right)}\right)}^{-1}\right]}_{u,u}^{-1}$$

To accommodate within‐species variation when only summary data are available, the above algorithm is identical except that in steps 1, **T** ^(*e*)^ is replaced with **T** ^(*e*)^ + **B** ^(*e*)^ where **B** ^(*e*)^ is an estimate of within‐species covariance (e.g., a diagonal matrix with squared standard errors) (Ives et al., 2007). For species mean imputation in step 5, **B** ^(*e*)^ is *not* added to **T** ^(*e*)^ (Bruggeman et al., 2009; Goolsby et al., 2017).

To accommodate within‐species variation when raw data are available, the algorithm is nearly identical as above except that initialization (step 1) and imputation of missing data (step 5) is performed on raw data (i.e., an individual within‐species observation) rather than on species means, and **T** ^(*e*)^ replaced entirely with **B** ^(*e*)^ in steps 1 and 5. **B** ^(*e*)^ may be set to an a priori determined value (Ives et al., 2007) or jointly estimated during maximum likelihood optimization (Felsenstein, 2008). Typically, **B** ^(*e*)^ is assumed to be identical across species if **B** ^(*e*)^ is to be estimated via numerical optimization (Felsenstein, 2008). Steps 2–4 proceed as normal, except that species nodes are treated as “internal nodes” since the “tips” of the tree are individual observations, and hence edges giving rise to species nodes are included in the postorder and preorder recursion steps. When *e* gives rise to a species node, step 4 provides estimates of species means, and step 5 provides raw data imputations for missing values.

## 3 Results and Discussion

### 3.1 R implementation

The proposed ancestral state reconstruction algorithm is implemented in the R package *Rphylopars* (Goolsby et al., 2017). For simple Brownian motion evolution on univariate or multivariate data, maximum likelihood ancestral states and confidence intervals may be fit using the *Rphylopars* function *anc.recon*. For more complex models with missing data, within‐species variation, or alternative evolutionary model specifications (e.g., Ornstein–Uhlenbeck or Early‐Burst), the *Rphylopars* function *phylopars* must be used to fit evolutionary model parameters, which are then used to compute maximum likelihood ancestral states using the fast algorithm.

### 3.2 Speed comparisons: univariate data

Here, we compare the speed of the proposed algorithm is implemented in *anc.recon* with four standard methods as implemented in R for performing ML ancestral state reconstruction: (1) numerical optimization (*ace* function in the R package *ape*, Paradis et al., 2004), (2) generalized least squares with direct matrix inversion (Martins & Hansen, 1997), (3) generalized least squares avoiding matrix inversion using the linear‐time algorithm described in Ho and Ané (2014), and (4) the rerooting method implemented in the *fastAnc* function in the *phytools* package (Revell, 2012). Univariate traits were simulated on phylogenies of size 32, 64, 128, 256, 512, 1,024, 2,048, and 4,096 species using the *rTraitCont* and *rtree* functions in *ape* (Paradis et al., 2004). For each tree size, five simulated phylogenies and datasets were generated, and the mean and standard deviation of computation time was recorded for each method. In order to be able to distinguish the computation time of the algorithm described here from 0 s (using the *system.time* function, which has a resolution of 10 ms), speed assessments using *anc.recon* were performed on 1,000 replicated function calls and the total computation time was subsequently divided by 1,000.

**Figure 1:**
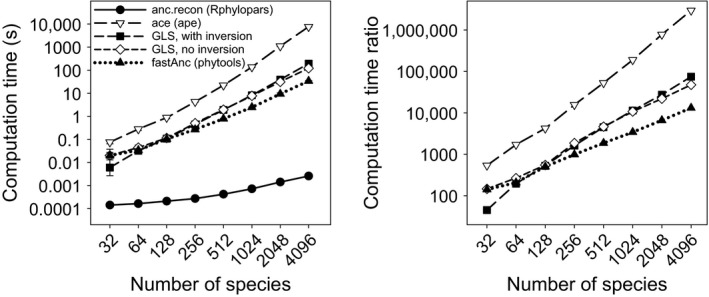
Computation times (left) for univariate ancestral state reconstruction using the described fast two‐pass algorithm (*anc.recon* function, *Rphylopars* package), numerical optimization (*ace* function, *ape* package), generalized least squares (GLS) with matrix inversion (Martins & Hansen, 1997), GLS without matrix inversion (Ho & Ané, 2014), and the rerooting method implemented in the *fastAnc* function in *phytools*. The right panel consists of ratios of computation times for optimization, GLS with and without inversion, and rerooting relative to the described fast algorithm. All *anc.recon* run times completed in fewer than 10 ms, whereas the next‐fastest method (*fastAnc*) ran from 141 to 13,104 times slower than *anc.recon*, and the slowest method (*ace*) ranged from 537 to nearly three million times slower than *anc.recon* (right panel). Error bars (where visible) indicate standard deviation of five replicate runs per simulated number of species

For all simulated datasets, *anc.recon* computation time was below 10 ms, whereas the *fastanc* function took up to 36 s for the largest simulated dataset (4,096 taxa), with a polynomial increase in computation time as the number of species increased (Figure 1a). Other methods were even slower, including numerical optimization, in which *anc.recon* performed approximately 3,000,000 times faster than *ace* (Figure 1b). Even on the smallest simulated datasets (32 taxa), *anc.recon* was approximately 140 times faster than *fastAnc* (the next fastest method), and for the largest dataset, *anc.recon* was over 13,000 times faster than *fastAnc*. Additionally, a decrease in precision was observed for numerical optimization in the *ace* function, something not shared by the method described here (which algorithmically computes exact maximum likelihood estimates). Speed assessments were also performed using only *anc.recon* on phylogenies ranging from 256 to 2,097,152 (2^8^ to 2^21^) taxa, the largest of which completed in fewer than 3 s. Across all simulations, *anc.recon* exhibited linear increases in computation time (Table 1). R code for performing the simulations used to generate all figures is supplied in Appendix S1.

**Table 1:** Mean computation times for *anc.recon* ancestral state reconstruction on univariate datasets with 256 to 2,097,152 (2^8^ to 2^21^) species. For each number of species, five simulated phylogenies and datasets were generated

Number of species	Computation time (s)	Standard deviation
256	0.0003	1.87E‐05
512	0.0004	1.67E‐05
1,024	0.0007	1.14E‐05
2,048	0.001	3.36E‐05
4,096	0.003	8.29E‐05
8,192	0.006	0.0004
16,384	0.011	0.0006
32,768	0.021	0.0004
65,536	0.052	0.0084
131,072	0.110	0.0071
262,144	0.222	0.0148
524,288	0.520	0.0418
1,048,576	1.136	0.0929
2,097,152	2.422	0.4268

## 4 Conclusion

The algorithm described here generalizes existing efficient algorithms (Elliot, 2015; Felsenstein, 2004; Maddison, 1991) and is capable of performing maximum likelihood ancestral state reconstruction on phylogenies containing one million taxa in fewer than 2 s, using modest computational resources (i.e., a standard laptop). The method can be expanded to incorporate a variety of models, including multivariate generalizations, within‐species variation, non‐Brownian evolutionary models, rate heterogeneity, and more. As the number of taxa in phylogenetic comparative studies continues to rise, efficient linear‐time algorithms will become increasingly critical. Additionally, frameworks requiring thousands or millions of repeated calculations, such as parametric bootstrapping and Bayesian analyses, will also benefit from the continued improvement of fast algorithms.

## Conflict of Interest

None declared.
